# Suspected Labour as a Reason for Emergency Medical Services Team Interventions in Poland—A Retrospective Analysis

**DOI:** 10.3390/healthcare10010049

**Published:** 2021-12-28

**Authors:** Ewa Rzońca, Agnieszka Bień, Arkadiusz Wejnarski, Joanna Gotlib, Grażyna Bączek, Robert Gałązkowski, Patryk Rzońca

**Affiliations:** 1 Department of Obstetrics and Gynecology Didactics, Faculty of Health Sciences, Medical University of Warsaw, 00-575 Warsaw, Poland; gbaczek@wum.edu.pl; 2 Chair and Department of Development in Midwifery, Faculty of Health Sciences, Medical University of Lublin, 20-081 Lublin, Poland; agnieszka.bien@umlub.pl; 3 Faculty of Medical Sciences and Health Sciences, Siedlce University of Natural Sciences and Humanities, 08-110 Siedlce, Poland; arkadiusz.wejnarski@uph.edu.pl; 4 Department of Education and Research in Health Sciences, Faculty of Health Sciences, Medical University of Warsaw, 02-091 Warsaw, Poland; joanna.gotlib@wum.edu.pl; 5 Department of Emergency Medical Services, Faculty of Health Sciences, Medical University of Warsaw, 00-575 Warsaw, Poland; robert.galazkowski@wum.edu.pl; 6 Department of Human Anatomy, Faculty of Health Sciences, Medical University of Warsaw, 02-004 Warsaw, Poland; przonca@wum.edu.pl

**Keywords:** Emergency Medical Service, pregnant women, labour, health care

## Abstract

The purpose of this study was to present the characteristics of Emergency Medical Services (EMS) team responses to calls regarding suspected labour in out-of-hospital settings in Poland. We performed a retrospective analysis of EMS team interventions in cases of suspected onset of labour outside a hospital setting. The analysis included 12,816 EMS team responses to calls regarding women in suspected labour in the period between January 2018 and December 2019. The mean age of the patients studied was 28.24 years (SD = 6.47). The majority of patients were at term (76.36%) and in their second pregnancy (29.96%). EMS teams were most often dispatched in the summer (25.95%) and in urban areas (63.26%). Most EMS teams were basic (68.99%) and interventions most often took place between 19:00 and 06:59 (63.14%). Significant differences were observed between preterm and term pregnant women attended by EMS teams in terms of variables such as the age of the patient, number of previous labours, history of miscarriage, presence of vaginal bleeding, time of year, location of call, type and composition of EMS team dispatched, urgency code and time of call, duration of intervention, selected emergency medical procedures performed and test results.

## 1. Introduction

Pregnancy and labour are extraordinary and unique experiences that have a multifaceted impact on a woman’s life and the lives of her loved ones [1,2,3,4]. Pregnancy and childbirth involve a variety of emotions, expectations and plans. Expectant women take steps to ensure they are provided with the best possible professional medical care during this critical period [3,4,5,6,7,8,9]. When preparing for labour, women draw up a birth plan, which is a written document with information about their chosen birth setting as well as preferences and expectations regarding labour and the care provided to mother and baby after birth [10,11]. Researchers undertake multidimensional studies on different aspects of childbirth, including those related to birth settings, e.g., a hospital or birth centre or home (home birth), in order to better understand the choices of women in this process [12,13,14,15,16,17].

In Poland, perinatal care is primarily provided by obstetrician–gynaecologists and less frequently by midwives. Most births take place in a hospital setting. The option of home birth is not available under any public prenatal care or motherhood support programme, with home birth services being provided by a small group of independent midwives [18]. Moreover, the Polish public believes that hospitals are safest birth setting, as they offer the necessary facilities and resources to provide essential medical procedures and interventions [13]. Women who give birth in hospital are assisted by a midwife, who is responsible for delivering the baby. When any pathological complication occurs, the midwife immediately hands over the care of the labouring patient to an obstetrician and assists them during the labour and delivery [19].

The unpredictable nature of childbirth occasionally results in unplanned out-of-hospital births or births before the arrival of pre-organized assistance [20,21]. The Polish Emergency Medical Services were established to provide care to patients with life or health threatening emergencies. This system includes basic structures such as hospital emergency departments and EMS teams, as well as advanced units e.g., Helicopter Emergency Medical Services (HEMS) teams. The task of both EMS and HEMS teams is to deliver emergency medical care on site and transport the patient to the hospital. EMS teams may include physicians, emergency medical technicians, and nurses. It should be noted that Polish EMS teams are classified as basic (non-physician-staffed) or specialist (physician-staffed). In terms of the number of responders per team, there can be either two-person or three-person EMS teams. In Poland, the basic (non-physician-staffed) EMS teams can include two or three members, whereas specialist (physician-staffed) teams always include three members [22].

This study aims to present the characteristics of EMS team interventions in cases of suspected labour in Poland.

## 2. Materials and Methods

**Figure 1:**
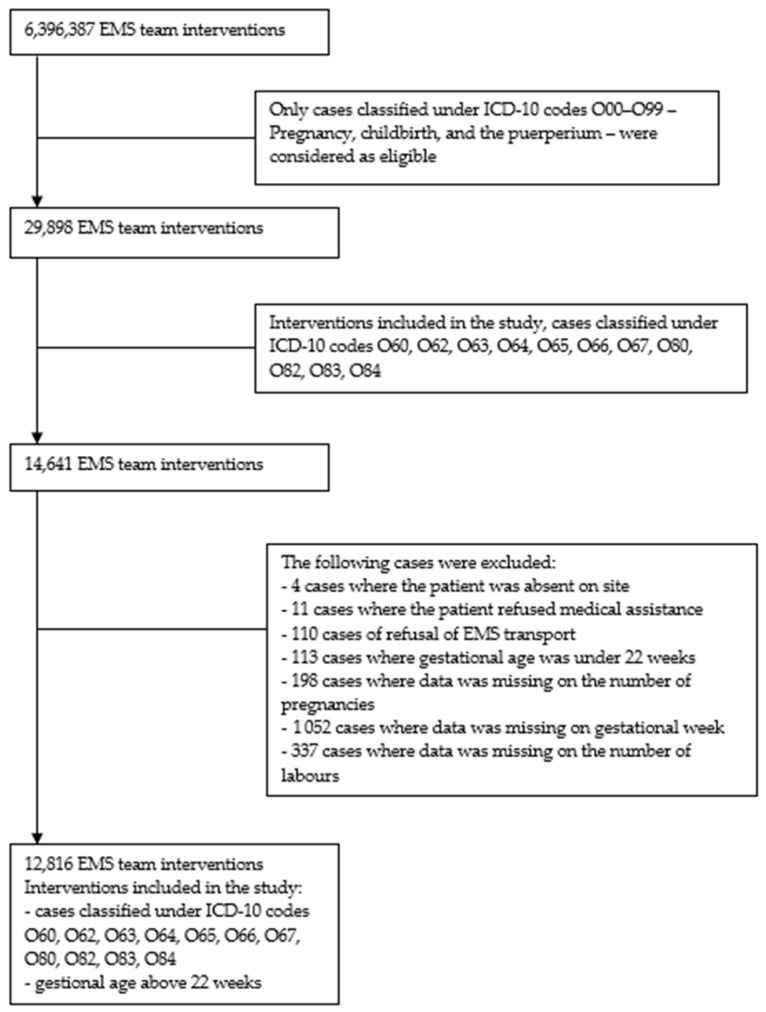
Flowchart of the recruitment process of EMS team interventions in cases of suspected labour (N = 12,816).

The study was based on a retrospective analysis of interventions by basic and specialist EMS teams in cases of suspected onset of labour in out-of-hospital settings in Poland. The study period was from January 2018 to December 2019 and was conducted using data obtained from a database of Poland’s National Monitoring Centre of Emergency Medical Services. This is an information and communication system that is used for accepting emergency calls and event notifications from emergency numbers, dispatching EMS teams, preparing medical records, and managing these calls and events [23]. Data obtained from the database included emergency medical procedure and EMS team dispatch records. The documentation was analysed to obtain the following information: date and location of call; details of the labouring patient—the woman’s age, obstetric history (gestational week, number of pregnancies and deliveries, history of miscarriage, contractions, bleeding, status of the membranes), main diagnoses based on the International Statistical Classification of Diseases and Related Health Problems (ICD—10); patient clinical parameters; emergency medical procedures performed; other characteristics of the intervention. The study included all cases classified by the EMS team under ICD-10 codes O60, O62, O63, O64, O65, O66, O67, O80, O82, O83 and O84 where the gestational age was above 22 weeks. Exclusion criteria were refusal of medical assistance, cancellation of call, absence of the patient on site and gestational age of less than 22 weeks. Out of the 6,396,387 EMS team interventions in Poland in the period analysed, 12,816 EMS team interventions were selected for the final analysis (Figure 1).

This study was approved by the Polish Ministry of Health. The study protocol was submitted to the Bioethics Committee at the Medical University of Warsaw, which confirmed that this study did not require consent due to its retrospective nature.

The data obtained from the documentation analysis were analysed statistically using the STATISTICA software, version 13.2 (Tibco Software Inc., Palo Alto, CA, USA). Qualitative data were described using numbers (*n*) and percentages (%), while quantitative data were reported using means (M) or medians (Me) and standard deviations (SD) or interquartile ranges (IQRs). Distribution normality for quantitative variables was tested using the Kolmogorov–Smirnov test and the Lilliefors test. Statistically significant differences between qualitative variables were tested using the Chi^2^ test, and differences between two independent groups were tested using the non-parametric Mann–Whitney U-test. The impacts of particular variables (predictors) on the duration of pregnancy were examined with logistic regression using stepwise selection. A *p* < 0.05 was used to indicate significance, with the statistical power at minimum 80% (0.8).

## 3. Results

The mean age of the pregnant patients studied was 28.24 years (SD = 6.47). Most patients were at term (76.59%), in their second pregnancy (29.69%), had not given birth before (32.58%), had an uncomplicated obstetric history (86.71%), had contractions (81.84%) and had rupture of membranes (53.14%). Most patients did not have vaginal bleeding (84.92%) (Table 1).

Table 2 shows the characteristics of EMS team responses to calls regarding suspected labour in Poland. EMS teams were most commonly dispatched in the summer (26.00%), in urban areas (62.97%), with urgency code 2 (meaning that an available EMS team must respond to a health emergency requiring emergency medical assistance) (54.39%) and between 19:00 and 06:59 (63.26%). Most were basic (69.98%) two-person (59.83%) teams. The emergency call was most commonly made by the labouring patient herself (23.88%). The mean duration of EMS team intervention in cases of suspected labour was 34 min.

Emergency medical procedures most commonly performed by EMS team members for labouring patients were pulse oximetry (74.72%), the taking of medical history (74.72%) and blood pressure measurement (71.53%). Details regarding the emergency medical procedures performed and selected physical examination findings are shown in Table 3.

Table 4 shows an analysis of associations between duration of pregnancy and selected variables regarding the patients and EMS team responses to calls. Our analysis shows that women with preterm pregnancies attended by EMS teams were more likely to be slightly older than patients at term (28.71 vs. 28.10 years). Preterm patients were more likely to have never given birth before, have a history of miscarriage and have vaginal bleeding. EMS teams were more often dispatched to patients with preterm pregnancies in the summer and spring and in urban areas. EMS teams attending those patients were usually specialist teams, composed of three persons and were most often dispatched with urgency code 1, between 07:00 and 18:59. The duration of intervention was significantly longer in cases of preterm women than in cases of women with term pregnancies (39 vs. 36 min). All of these differences were statistically significant (*p* < 0.05).

Our analysis demonstrated that emergency medical procedures such as pulse oximetry, blood pressure measurement, physical examination, capillary blood glucose measurement, intravenous cannulation and intravenous administration of medication were more often performed by EMS teams in cases of patients with preterm pregnancies than in patients at term. We found that patients with preterm pregnancies were more likely than patients with term pregnancies to have a higher heart rate, pallor and abdominal pain, as confirmed by physical examination, whereas patients with term pregnancies had higher systolic and diastolic blood pressure values compared with preterm patients. These differences were also all statistically significant (Table 5).

Table 6 shows the results of regression analysis performed using the Enter regression method for the ‘duration of pregnancy’–‘preterm pregnancy’ variable. The model explains 17.2% of variation for the ‘duration of pregnancy’ variable. Statistically significant predictors in this model were the number of labours (β = 0.135; *p* < 0.05), age of the pregnant patient (β = −0.050; *p* < 0.001), location of call (β = 0.021; *p* < 0.05) and number of pregnancies (β = −0.092; *p* < 0.05). The analysis revealed that the number of labours (β = 0.135) and the number of pregnancies (β = −0.092) had the strongest impact on the risk of preterm birth. Our analysis of the direction of association between particular variables indicated that the risk of preterm birth is greater when the number of previous labours is higher and in patients calling from urban areas. Moreover, the analysis demonstrated that the fewer the number of previous pregnancies and the younger the patient, the higher the risk of preterm birth. A history of miscarriage was not found to be statistically significant in this model (β = 0.031; *p* > 0.05).

## 4. Discussion

Childbirth is unpredictable and may sometimes occur in an unplanned setting, in which case medical assistance by EMS personnel may be required [1,2,20,21,24]. The complex and multifaceted nature of childbirth motivated the present study, the aim of which was to present the characteristics of EMS team interventions for women in suspected labour in Poland.

Unplanned out-of-hospital births can be defined as labours without the presence of a midwife and without medical care provided, which normally create optimum conditions for childbirth [25]. Unplanned out-of-hospital births and births before arrival (BBA) are of interest to researchers around the world. Such births are associated with adverse perinatal outcomes or increased mortality [26,27]. Findings from previous studies indicate that the mean age of the women studied who had such a birth was 30 years [25,28,29,30] and that most of the patients were multiparous [25,28,29,30,31,32] and delivered at term [29,30,31,32]. In contrast, in a study by Strehlow et al. (2016) on the characteristics of women in their third trimester of pregnancy using Emergency Medical Services for pregnancy-related problems in India, the median age of the patients studied was 23 years, which is lower compared with the studies cited above [31], while in a study by Pasternak et al. (2018) on perinatal outcomes in unplanned out-of-hospital births in Israel, the mean age of the patients studied was higher, i.e., 35 years [32]. In their study, Javaudin et al. (2019) found that emergency calls for patients in labour were most often made by persons close to them [30]. In our present study, the mean age of the patients studied was 28 years. Most patients were at term and in their second pregnancy, and emergency calls were most often made by the patient herself. Varying results of studies conducted in different countries and pertaining to the mean age of the women studied indicate that age is not a determinant of out-of-hospital births.

The characteristics of interventions by EMS and HEMS teams vary significantly depending on the type of health problem or life threatening emergency they relate to [33,34,35,36]. An analysis of calls from emergency numbers in Copenhagen carried out by Møller et al. (2015) demonstrated that the number of emergency calls was highest in the winter and during daytime [33], while findings from a study on the characteristics of Helicopter Emergency Medical Service missions in rural areas in Poland indicate that HEMS teams are more often dispatched in the summer [34]. In their study, Faramand et al. (2020) found that interventions involving patient transport most often took place in the autumn [35]. Wang et al. (2013) analysed interventions by EMS teams from 29 states of the USA and found that the vast majority of interventions took place between 15:00 and 22:59 [36]. For emergencies regarding suspected labour in out-of-hospital settings, it was found that EMS teams were most often dispatched to patients in suspected labour in the summer, during night time. These data indicate that the needs for EMS and HEMS interventions vary, irrespective of the time of the year. However, it should be stressed that situations in which EMS teams are in attendance at a birth are rare [24]. Our findings demonstrated that most interventions by basic EMS teams in cases of suspected labour concerned patients with term pregnancies, while patients with suspected preterm labour were most often attended by three-person specialist EMS teams dispatched with urgency code 1. This can be explained by the nature of preterm birth and its serious consequences for life-long health [37,38,39,40]. An analysis of our findings also demonstrated that the duration of EMS team interventions in cases of suspected preterm labour was longer than that of interventions concerning patients with term pregnancies. These data indicate that the complexity of a given case translates into the duration of EMS team interventions.

Strehlow et al. (2016) studied emergency medical technician interventions concerning women in their third trimester of pregnancy and found that emergency medical technicians consistently measured the basic vitals of their patients and positioned them in the left lateral position [31]. A study by McLelland et al. (2018) demonstrated that the medical procedure most often performed by paramedics for patients in labour was the administration of medication—analgesics (methoxyflurane) [20]. An analysis of our findings revealed that the EMS team members responding to calls relating to women in suspected labour performed emergency medical procedures for the patients in only a small percentage of cases. Moreover, data gaps in the medical records were identified. It can be assumed that emergency medical procedures were in fact performed in a greater number of cases, but they were not recorded in the medical records, as the patients had to be quickly transported to a hospital, which indicates a serious gap in the healthcare system. The observations made by McLelland et al. (2018) in their study are also important in this respect, including the observation that all information is given by the pregnant patient voluntarily and therefore paramedics can only document the information with which the patient provides them [20]. It should be noted that assistance with the delivery of a baby is a relatively rare event in the daily practice of EMS teams [24,41,42], as also confirmed by our findings.

In this study, we further analysed the association between the duration of pregnancy and the characteristics of patients in suspected labour and the procedures performed for the patients by EMS teams. It should be noted that all pregnant women between 22 and 37 weeks of gestation are at theoretical risk of preterm birth [37,38]. Preterm birth and the resulting prematurity of the infant and its numerous consequences are problematic and challenging not only for the baby and their family, but also for public health, in the broad sense of governments and healthcare systems [37,38,39,40]. These issues are associated not only with greater mortality, but also with increased morbidity and healthcare costs [43]. There are numerous risk factors for preterm labour, including a history of preterm birth, the age of the mother, vaginal bleeding, multiple gestations, infections, stress, low pregnancy body mass index, premature dilatation and shortening of the cervix and abnormal pelvic anatomy [40,44,45,46]. Our findings indicated that the factors having an impact on the risk of preterm labour in this study were the number of pregnancies, number of previous labours, the age of the patient and the location of the call regarding suspected labour. Bills et al. (2018) published a study on decreases in the early neonatal mortality rate in India in the context of EMS use by pregnant women. The authors found that the most common reasons for the emergency calls were abdominal pain and spasm, rupture of membranes, contractions, and in significantly fewer cases, vaginal bleeding [47]. In their study, Strehlow et al. (2016) found that EMS teams were most often dispatched to pregnant patients in their third trimester living in rural areas, the vast majority of whom had contractions [31]. Combier et al. (2020) carried out a nationwide population-based study on unplanned out-of-maternity deliveries (OMD) in France. They found that women who delivered out-of-maternity had a higher risk of delivering before 37 weeks of gestation and that the risk of out-of-maternity delivery decreased for patients living in urban centres and in their surrounding suburbs [48]. In conclusion to their study, Pasternak et al. (2018) noted that women with no caesarean deliveries and operative deliveries in their obstetric history are at greatest risk of unplanned out-of-hospital birth [32]. An analysis of our findings demonstrated that most of the preterm patients studied had not given birth before. Moreover, preterm patients were more likely to have a complicated obstetric history (miscarriage) and were more likely than patients at term to experience vaginal bleeding. Interventions concerning women in suspected preterm labour most often took place in urban areas, in warm seasons (spring, summer), during the daytime. It needs to be emphasised that other researches have demonstrated the impact of the environment on the pregnancy course to be one of the risk factors for preterm labour. They underline the negative effect of air pollution, more often observed in urban areas, additionally exacerbated by traffic intensity, mainly during the day, which might provide justification for the results we obtained [49,50].

This study is important because it reports the first analysis of Polish EMS team interventions in cases of women in suspected labour. These teams are a key component in the healthcare system by providing on-site professional assistance to patients. The study involved an analysis of all calls registered by Poland’s National Monitoring Centre of Emergency Medical from January 2018 to December 2019, which constitutes its strength and allowed for gathering reliable data on EMS team interventions in cases of suspected labour. The strength of our work is that it was a retrospective study. Thus, it was possible to conduct a detailed analysis of the data contained in the medical records of EMS. However, the present study also has certain limitations. The analysis only included information contained in the EMS documentation, i.e., emergency medical procedures and EMS team dispatch records. However, we do not have information about whether a given call attended by the EMS team dispatched ended with a birth. Furthermore, we do not have information on subsequent patient management, obstetric outcome or the health of the patient and the foetus or newborn baby. These limitations do not, however, impair the quality of the study, they only indicate directions for further research.

The strengths and limitations of our study indicate the need for further research in this area. For example, it seems necessary to explore the reasons why Polish women in suspected labour call EMS. Moreover, it needs to be emphasised that attending births by members of EMS teams is not part of their everyday practice. Therefore, it is important to know how calls regarding suspected labour are perceived by the members of EMS teams. Further studies on out-of-hospital births are necessary to provide a better understanding of the subject as well as to ensure that EMS teams are better prepared to attend births and deliver the best possible and highest quality care to labouring patients and their newborn.

## 5. Conclusions

The mean age of patients with suspected labour attended by EMS teams was 28 years. The patients tended to be multiparous, at term and have contractions. Significant differences were observed between preterm, and term pregnant women attended by EMS teams in terms of variables such as the age of the patient, the number of previous labours, history of miscarriage, presence of vaginal bleeding, time of year, location of call, type and composition of the EMS team dispatched, urgency code and time of call, duration of intervention, selected emergency medical procedures performed and test results. The factors having an impact on the risk of preterm labour in the women studied were the numbers of pregnancies and labours, the age of the patient and the location of call.

The results of our study indicate that the complexity of a given case—suspected labour or term labour in out-of-hospital settings—translates into the way the EMS team intervention is carried out. At the same time, it is necessary to conduct further research on EMS team interventions in cases of suspected labour, so that EMS team members can provide women in labour with the best quality of care, as these professionals are a crucial part of the healthcare system, offering professional assistance on site.

## Figures and Tables

**Table 1:** Characteristics of pregnant patients attended by EMS teams.

Age M (SD)	28.24 (6.47)
Duration of pregnancy *n* (%)	
preterm pregnancy	3000 (23.41)
term pregnancy	9816 (76.59)
Gestational week M (SD)	37.87 (3.14)
Number of pregnancies *n* (%)	
1st	3588 (28.00)
2nd	3805 (29.69)
3rd	2420 (18.88)
4th	1452 (11.33)
5th or subsequent	1551 (12.10)
Number of pregnancies Me (IQR)	2 (1–3)
Number of labours *n* (%)	
1st	4175 (32.58)
2nd	3883 (30.30)
3rd	2301 (17.95)
4th	1237 (9.65)
5th or subsequent	1220 (9.52)
Number of labours Me (IQR)	2 (1–3)
History of miscarriage *n* (%)	
yes	1703 (13.29)
no	11,113 (86.71)
Contractions *n* (%)	
yes	10,489 (81.84)
no	2327 (18.16)
Membranes *n* (%)	
intact	3857 (30.10)
ruptured	8959 (69.90)
Bleeding *n* (%)	
yes	779 (6.08)
no	10,882 (84.92)
no examination performed	1155 (9.00)

**Table 2:** Characteristics of EMS (Emergency Medical Services) team responses to calls regarding suspected labour.

Time of year *n* (%)	
winter	3300 (25.75)
spring	3069 (23.95)
summer	3332 (26.00)
fall	3115 (24.30)
Location of call *n* (%)	
urban area	8070 (62.97)
rural area	4746 (37.03)
EMS team type *n* (%)	
basic	8968 (69.98)
specialist	3848 (30.02)
EMS team composition *n* (%)	
two-person	7668 (59.83)
three-person	5148 (40.17)
Urgency code *n* (%)	
code 1 ^1^	5845 (45.61)
code 2 ^2^	6971 (54.39)
Time of call *n* (%)	
07:00–18:59	4709 (36.74)
19:00–06:59	8107 (63.26)
Caller *n* (%)	
labouring patient	3061 (23.88)
family	1607 (12.54)
other	842 (6.57)
unidentified	7306 (57.01)
Duration of intervention Me (IQR)	34 (24–46)

^1^ Code 1—an EMS team with the shortest estimated time to reach the scene must respond immediately to a health emergency requiring immediate emergency medical assistance; ^2^ code 2—an available EMS team must respond to a health emergency requiring emergency medical assistance.

**Table 3:** Characteristics of the emergency medical procedures performed by EMS teams and selected physical examination findings.

**Emergency Medical Procedures *n* (%)**	
taking of medical history	9576 (74.72)
pulse oximetry	9576 (74.72)
blood pressure measurement	9167 (71.53)
physical examination	8363 (65.25)
vital parameter monitoring	8333 (65.02)
intravenous cannulation	2145 (16.74)
blood glucose measurement	1531 (11.95)
gynaecological examination	763 (5.95)
manual assistance during a spontaneous delivery	262 (2.04)
intravenous medication	259 (2.02)
**Physical Examination Findings**	
Saturation % M (SD)	97.95 (1.16)
Systolic blood pressure mmHg M (SD)	128.42 (15.86)
Diastolic blood pressure mmHg M (SD)	78.93 (10.31)
Heart rate beat/min M (SD)	92.44 (20.39)
Blood glucose mg/dL M (SD)	105.33 (24.52)

**Table 4:** Analysis of associations between duration of pregnancy and selected variables.

Variables	Preterm Pregnancy	Term Pregnancy	*p*-Value
Age M (SD)	28.71 (6.53)	28.10 (6.45)	0.0000 **
Number of labours *n* (%)			0.0438 *
1	1040 (34.67)	3135 (31.94)	
2	872 (29.07)	3011 (30.67)	
3	524 (17.47)	1777 (18.10)	
4	298 (9.93)	939 (9.57)	
5 and more	266 (8.87)	945 (9.72)	
History of miscarriage *n* (%)			0.0000 *
yes	494 (16.47)	1209 (12.32)	
no	2506 (83.53)	8607 (87.68)	
Bleeding *n* (%)			0.0000 *
yes	286 (9.53)	493 (5.02)	
no	2448 (81.60)	8434 (85.92)	
no examination performed	266 (8.87)	889 (9.06)	
Time of year *n* (%)			0.0176 *
winter	713 (23.77)	2587 (26.35)	
spring	754 (25.13)	2315 (23.58)	
summer	812 (27.07)	2520 (25.67)	
fall	721 (24.03)	2394 (24.39)	
Location of call *n* (%)			0.0139 *
urban area	1946 (64.87)	6124 (62.39)	
rural area	1054 (35.13)	3692 (37.61)	
EMS team type *n* (%)			0.0000 *
basic	1984 (66.13)	6984 (71.15)	
specialist	1016 (33.87)	2832 (28.85)	
EMS team composition *n* (%)			0.0000 *
2-person	1668 (55.60)	6000 (61.12)	
3-person	1332 (44.40)	3816 (38.88)	
Urgency code *n* (%)			0.0173 *
code 1	1530 (51.00)	4315 (43.96)	
code 2	1470 (49.00)	5501 (56.04)	
Time of call *n* (%)			0.0000 *
07:00–18:59	1257 (41.90)	3452 (35.17)	
19:00–06:59	1743 (58.10)	6364 (64.83)	
Duration of intervention Me (IQR)	39 (25-36)	36 (24-33)	0.0000 **

* The Chi^2^ test; ** the Mann–Whitney U-test.

**Table 5:** Analysis of associations between duration of pregnancy and the emergency medical procedures performed and selected physical examination findings.

Variables	Preterm Pregnancy	Term Pregnancy	*p*-Value
Pulse oximetry *n* (%)			0.0371 *
yes	2285 (76.17)	7291 (74.28)	
no	715 (23.83)	2525 (25.72)	
Blood pressure measurement *n* (%)			0.0001 *
yes	2232 (74.40)	6935 (70.65)	
no	768 (25.60)	2881 (29.35)	
Physical examination *n* (%)			0.0021 *
yes	2028 (67.60)	6335 (64.54)	
no	972 (32.40)	3481 (35.46)	
Capillary blood glucose measurement *n* (%)			0.0002 *
yes	417 (13.90)	1114 (11.35)	
no	2583 (86.10)	8702 (88.65)	
Intravenous medication *n* (%)			0.0000 *
yes	123 (4.10)	136 (1.39)	
no	2877 (95.90)	9680 (98.61)	
Intravenous cannulation *n* (%)			0.0000 *
yes	633 (21.10)	1512 (15.40)	
no	2367 (78.90)	8304 (84.60)	
Systolic blood pressure mmHg M (SD)	127.25 (16.40)	128.79 (15.66)	0.0000 **
Diastolic blood pressure mmHg M (SD)	78.48 (10.59)	79.07 (10.21)	0.0175 **
Heart rate beat/min M (SD)	93.67 (20.17)	92.06 (20.44)	0.0000 **

* The Chi^2^ test; ** the Mann–Whitney U-test.

**Table 6:** Logistic regression analysis for the ‘duration of pregnancy’ variable.

Selected Predictors	Duration of Pregnancy Adjusted R^2^ = 0.172 F = 19.150 *p* < 0.001			
	B	β	*t*	*p*
History of miscarriage	0.037	0.031	1.800	>0.05
Number of labours	0.037	0.135	3.089	<0.05
Age of the pregnant patient	−0.003	−0.050	−4.756	<0.001
Location of call	0.018	0.021	2.266	<0.05
Number of pregnancies	−0.024	−0.092	−1.973	<0.05

## Data Availability

The data presented in this study are available on request from the corresponding author.
